# Supplementary material for: Towards New Scaffolds for Antimicrobial Activity—In Silico/In Vitro Workflow Introducing New Lead Compounds
Source: Antibiotics (Basel). 2024 Dec 27;14(1):11. doi: 10.3390/antibiotics14010011 (PMC11763081; doi:10.3390/antibiotics14010011)
Supplement: Supplementary file 1 [file antibiotics-14-00011-s001.zip › Supplementary Table S1.pdf]

Supplementary Table

Table S1. Antibiotic susceptibility profiles of the isolates used in this study

Antibiotic	MIC (Interpretation)						
	S. aureus *ATCC 25923*	*MRSA* *2679:ST80*	*E. faecalis*	*K. pneumoniae*	*A. baumannii*	*E. coli* *ATCC 25922*	*P. aeruginosa* *ATCC 27853*
Amoxicillin /Clavulanate	**≤4/2 (S)**	**8/4 (R)**	**≤4/2 (S)**	**N/A**	**>32**	**N/A**	**N/A**
Ampicillin	**≤0.25 (S)**	**>8 (R)**	**≤4 (S)**	**>16 (R)**	**>16**	**≤8 (S)**	**>16**
Amikacin	**N/A**	**N/A**	**N/A**	**≤16 (S)**	**N/A**	**≤16 (S)**	**≤16 (S)**
Ampicillin/Sulbactam	**N/A**	**N/A**	**N/A**	**≤8/4 (S)**	**>16/8**	**≤8/4 (S)**	**>16/8**
Cefepime	**N/A**	**N/A**	**N/A**	**≤8 (S)**	**>16**	**≤8 (S)**	**≤8 (S)**
Cefotaxime	**N/A**	**N/A**	**N/A**	**≤1 (S)**	**>32**	**≤1 (S)**	**16**
Cefoxitin	**N/A**	**N/A**	**N/A**	**≤8 (S)**	**>8**	**≤8 (S)**	**>8**
Ceftazidime	**N/A**	**N/A**	**N/A**	**≤1 (S)**	**>16 (R)**	**≤1 (S)**	**4 (S)**
Cefuroxime	**N/A**	**N/A**	**N/A**	**≤4 (S)**	**>16**	**8 (S)**	**>16**
Ceftaroline	**≤0.5 (S)**	**≤0.5**	**N/A**	**N/A**	**N/A**	**N/A**	**N/A**
Ciprofloxacin	**≤1 (S)**	**≤1 (S)**	**≤2 (S)**	**≤1 (S)**	**>2**	**≤1 (S)**	**≤1 (S)**
Colistin	**N/A**	**N/A**	**N/A**	**≤2 (S)**	**N/A**	**≤2 (S)**	**4 (R)**
Clindamycin	**≤0.25 (S)**	**0.5**	**N/A**	**N/A**	**N/A**	**N/A**	**N/A**
Daptomycin	**≤1 (S)**	**≤1 (S)**	**N/A**	**N/A**	**N/A**	**N/A**	**N/A**
Erythromycin	**≤0.5 (S)**	**1 (S)**	**N/A**	**N/A**	**N/A**	**N/A**	**N/A**
Ertapenem	**N/A**	**N/A**	**N/A**	**≤0.5 (S)**	**>1**	**≤0.5 (S)**	**>1**
Fosfomycin	**≤32 (S)**	**≤32 (S)**	**≤32 (S**	**≤32 (S)**	**≤32**	**≤32 (S)**	**≤32**
Gentamicin	**8 (R)**	**≤1 (S)**	**≤8 (R)**	**≤4 (S)**	**8**	**≤4 (S)**	**≤4 (S)**
Imipenem	**N/A**	**N/A**	**N/A**	**≤1 (S)**	**>8**	**≤1 (S)**	**≤1 (S)**
Levofloxacin	**≤1 (S)**	**≤1 (S)**	**≤2 (S)**	**≤2 (S)**	**4**	**≤2 (S)**	**≤2(S)**
Linezolid	**4 (S)**	**>4 (R)**	**≤4 (S)**	**N/A**	**N/A**	**N/A**	**N/A**

Meropenem	**N/A**	**N/A**	**N/A**	**≤1 (S)**	**>8**	**≤1 (S)**	**≤1(S)**
Moxifloxacin	**N/A**	**N/A**	**N/A**	**≤0.5 (S)**	**>1**	**≤0.5 (S)**	**N/A**
Mupirocin	**≤256 (S)**	**≤256 (S)**	**N/A**	**N/A**	**N/A**	**N/A**	**N/A**
Nitrofurantoin	**≤32 (S)**	**≤32 (S)**	**≤32 (S)**	**≤32 (S)**	**>64**	**≤32 (S)**	**>64**
Oxacillin	**1**	**>2 (R)**	**N/A**	**N/A**	**N/A**	**N/A**	**N/A**
Penicillin	**≤0.12 (S)**	**>0.25 (R)**	**≤4 (S)**	**N/A**	**N/A**	**N/A**	**N/A**
Piperacillin	**N/A**	**N/A**	**≤4 (S)**	**>64 (R)**	**N/A**	**≤16 (S)**	**≤16 (S)**
Teicoplanin	**≤2 (S)**	**≤2 (S)**	**≤2 (S)**	**N/A**	**N/A**	**N/A**	**N/A**
Tetracycline	**≤1 (S)**	**>8 (R)**	**N/A**	**≤4 (S)**	**>8 (R)**	**≤4 (S)**	**>8**
Tigecycline	**N/A**	**N/A**	**N/A**	**≤1 (S)**	**N/A**	**≤1 (S)**	**N/A**
Tobramycin	**≤1 (S)**	**≤1 (S)**	**N/A**	**≤4 (S)**	**≤4**	**≤4 (S)**	**≤4 (S)**
Trimethoprim/ Sulfa	**≤2/38 (S)**	**≤2/38 (S)**	**N/A)**	**≤2/38 (S)**	**>4/76 (R)**	**≤2/38 (S)**	**>4/76**
Vancomycin	**2 (S)**	**2 (S)**	**2 (S)**	**N/A**	**N/A**	**N/A**	**N/A**

N/A: data not available; S: susceptible; R: resistant.
